# Effects of polygenic risk score and sodium and potassium intake on hypertension in Asians: A nationwide prospective cohort study

**DOI:** 10.1038/s41440-024-01784-7

**Published:** 2024-07-10

**Authors:** Eunjin Bae, Yunmi Ji, Jinyeon Jo, Yaerim Kim, Jung Pyo Lee, Sungho Won, Jeonghwan Lee

**Affiliations:** 1 Department of Internal Medicine, Gyeongsang National University Changwon Hospital, Changwon, Republic of Korea; 2 Department of Internal Medicine, College of Medicine, Gyeongsang National University, Jinju, Republic of Korea; 3 Institute of Medical Science, College of Medicine, Gyeongsang National University, Jinju, Republic of Korea; 4 College of Natural Sciences, Interdisciplinary Program in Bioinformatics, Seoul National University, Seoul, Republic of Korea; 5 Department of Public Health Sciences, Institute of Health & Environment, Seoul National University, Seoul, Republic of Korea; 6 Department of Internal Medicine, College of Medicine, Keimyung University School of Medicine, Daegu, Republic of Korea; 7 Department of Internal Medicine, Seoul National University Boramae Medical Center, Seoul, Republic of Korea; 8 Department of Internal Medicine, Seoul National University College of Medicine, Seoul, Republic of Korea; 9 Interdisciplinary Program for Bioinformatics, College of Natural Science, Seoul National University, Seoul, Republic of Korea; 10 RexSoft Corps, Seoul, Republic of Korea

**Keywords:** hypertension, polygenic risk score, sodium intake, potassium intake

## Abstract

Genetic factors, lifestyle, and diet have been shown to play important roles in the development of hypertension. Increased salt intake is an important risk factor for hypertension. However, research on the involvement of genetic factors in the relationship between salt intake and hypertension in Asians is lacking. We aimed to investigate the risk of hypertension in relation to sodium and potassium intake and the effects of genetic factors on their interactions. We used Korean Genome and Epidemiology Study data and calculated the polygenic risk score (PRS) for the effect of systolic and diastolic blood pressure (SBP and DBP). We also conducted multivariable logistic modeling to evaluate associations among incident hypertension, PRS_SBP_, PRS_DBP_, and sodium and potassium intake. In total, 41,351 subjects were included in the test set. The top 10% PRS_SBP_ group was the youngest of the three groups (bottom 10%, middle, top 10%), had the highest proportion of women, and had the highest body mass index, baseline BP, red meat intake, and alcohol consumption. The multivariable logistic regression model revealed the risk of hypertension was significantly associated with higher PRS_SBP_, higher sodium intake, and lower potassium intake. There was significant interaction between sodium intake and PRS_SBP_ for incident hypertension especially in sodium intake ≥2.0 g/day and PRS_SBP_ top 10% group (OR 1.27 (1.07–1.51), *P* = 0.007). Among patients at a high risk of incident hypertension due to sodium intake, lifestyle modifications and sodium restriction were especially important to prevent hypertension.

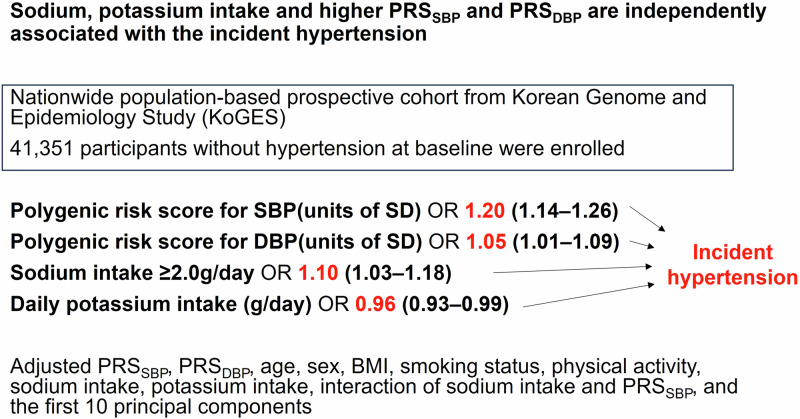

## Introduction

The number of adult patients aged 30–79 years with hypertension has rapidly increased to 1.28 billion worldwide, doubling in 30 years since 1990, and even the lowest prevalence has a high prevalence of over 17% [1]. In addition to being a representative risk factor for cardiovascular disease (CVD), hypertension itself is an important disease that should be prevented and treated since it can cause patient mortality [2, 3]. Hypertension is caused by both genetic and environmental factors, and its heritability is known to range from 30 to 60% [4–6]. Although a family history of hypertension provides easily accessible genetic information, it reflects indirect information about the overall genetic predisposition [7]. Recent studies have attempted to predict hypertension through polygenic risk scores (PRS), which calculate hypertension-related genomic variants using large-scale Genome-Wide Association Studies (GWAS) [8–11]. However, large-scale genetic data are required for PRS research. Therefore, most studies have been conducted only on Western populations, and studies on the influence of the systolic and diastolic blood pressure (SBP and DBP) PRS (PRS_SBP_ and PRS_DBP_) for hypertension in Asians are scarce. A study on PRS_SBP_ and hypertension in Asians has only been conducted in the Japanese population using the J-MICC (Japan Multi-Institutional Collaborative Cohort) study. This study showed that combinations of PRS for blood pressure and modifiable lifestyle factors such as smoking, drinking, sedentary lifestyle, and obesity were related to the prevalence of hypertension [11].

Lifestyle modifications such as sodium restriction, high consumption of vegetables and fruits, weight reduction, maintenance of an ideal body weight, and regular physical activity are important for prevention and treatment of hypertension [12]. Many previous studies on sodium and potassium intake have been conducted, and sodium restriction and high potassium intake in the diet are considered particularly important [13]. However, dietary control has a limited effect in preventing and treating hypertension, and there is no method for predicting and preparing for the risk of hypertension in each individual. For individualized prevention and treatment of hypertension, efforts should be made to identify find patient groups in which lifestyle modification and sodium restriction are particularly emphasized and patient groups with a genetic predisposition to hypertension.

Studies on the effects of PRS_SBP_, PRS_DBP_ on hypertension in Asians are lacking, and previous studies have not clarified whether dietary sodium or potassium intake are related to these genetic effects on the development of hypertension. Therefore, this large nationwide prospective cohort study aimed to investigate the association between PRS_SBP_, PRS_DBP_ and incident hypertension and its relationship with the effects of dietary sodium and potassium intake on the prevention of hypertension in Asians.

Point of view

- Clinical relevance
  It is important to note that genetic factors and high sodium intake are significant risk factors for the development of hypertension and that the risk of high sodium diet may be increased in patients with high genetic risk factors.
- Future direction:
  The effectiveness of reducing sodium intake to prevent the onset of hypertension in individuals with a high hereditary predisposition for hypertension needs to be verified.
- Consideration for the Asian population:
  The significant interaction between genetic factors and a high-salt diet, as well as the elevated risk of hypertension associated with genetic factors, may vary in other Asian countries and ethnic groups.

## Materials and methods

### Study design and populations

**Fig. 1:**
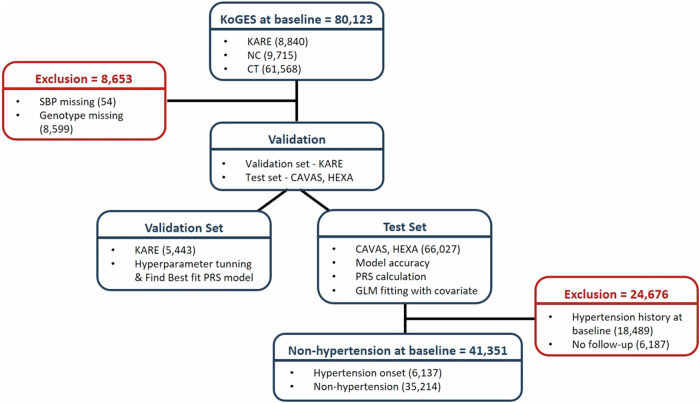
Flowchart of the study protocol

Data investigated in this study were collected from the Korean Genome and Epidemiology Study (KoGES) [14], a population-based prospective cohort that included participants aged ≥40 years from 2001 to 2013 and was composed of participants from the Korea Association Resource (KARE; *n* = 8,840), the Cardiovascular Disease Association Study (CAVAS; *n* = 9,715), and Health Examinee (HEXA; *n* = 61,568). Participants who had fewer than one SBP measurement (*n* = 54) and those whose genotype IDs did not match after quality control (*n* = 8599) were excluded. After exclusion, the KoGES cohort was split into two sets to validate the PRS model. The KARE cohort was designated as the validation set for tuning the hyperparameters and determining the best PRS (*n* = 5443). The CAVAS and HEXA cohorts were designated as the test set for PRS calculation and association analysis of PRS with hypertension (*n* = 66,027). Individuals with a history of hypertension at baseline (*n* = 18,489) and those without follow-up data (*n* = 6187) were excluded from the test set. Finally, 46,794 participants were enrolled from the three cohorts. The study design and quality control structure are summarized in Fig. 1.

### Primary outcome and definitions of variables

Hypertension was defined as a mean SBP ≥ 140 mmHg or mean DBP ≥ 90 mmHg, presence of a history of hypertension, or current use of medication for hypertension. In the test set, the primary outcome was defined as incident hypertension if participants were not classified as having hypertension at baseline but were classified as having hypertension at least once during the follow-up period (*n* = 6137).

The definitions of the variables considered as covariates are as follows: diabetes mellitus (DM) was defined by fasting glucose level ≥126 mmol/L, HbA1c ≥ 6.5%, a history of DM, or current use of medication for DM. Body mass index (BMI) was calculated as weight in kilograms divided by height in meters squared. If the SBP, DBP, waist circumference, or hip circumference were measured more than once, the average value of the measurements was used. Physical activity was defined as a response of “yes” to the question “Do you exercise regularly enough to make you sweat?” or moderate-to-vigorous physical activity for >30 min for ≥5 days per week. Daily total energy, sodium, and potassium intakes were calculated using a food intake frequency survey. Trained interviewers conducted the food intake frequency surveys using a semi-quantitative 103-item food frequency questionnaire, recording the frequency of consumption of each food item per participant over the past year. Nutrient intake was calculated as daily nutrient intake; the detailed methods for these calculations are described in the KoGES manual [15].

### Genotyping and imputation

KoGES samples were genotyped using the Korea Biobank Array (KoreanChip) [16] designed by the Center for Genome Science, Korea National Institute of Health (KNIH). Variants were identified using the K-medoid method [17]. Single-nucleotide polymorphisms (SNPs) were filtered out if (1) the missing genotype rate was >0.05, (2) Hardy–Weinberg equilibrium (HWE) *P*-value was <10^−^^5^, or (3) the SNPs were duplicated. Samples were also excluded if they showed (1) sex inconsistencies (0.2 <homozygosity chr X < 0.8), (2) missing genotype rate > 0.05, or (3) SNP heterozygosity rate> mean ± 3 × SD. Pre-phasing was performed using Eagle V2.4.1 [18] and imputation was conducted using Minimac4 [19] with a 1779 Northeast Asian reference panel [20]. After the imputation procedure, imputed SNPs with an imputation quality score (R^2^) < 0.3 and multi-allele characteristics were removed. Ultimately, 46,794 participants and 10,157,590 SNPs were analyzed. We used both PLINK v1.9 [21] and ONETOOL [22] for data quality control.

### PRS calculation (PRSsummary)

To derive the PRS, we used summary statistics from the BioBank Japan Project (BBJ), a hospital-based registry based on 136,597 individuals (http://jenger.riken.jp/en/) [23]. Because BBJ did not provide a GWAS result for hypertension, we utilized the GWAS result for SBP as a substitute. We considered SNPs with minor allele frequencies for BBJ larger than 0.005, 0.01, 0.05, or all SNPs.

The PRS for SBP and DBP were obtained using pruning+thresholding (P + T), clumping+thresholding (C + T) [24], LDpred-infinitesimal, LDpred-grid, LDpred-auto [25, 26], PRScs [27], and Lassosum-pseudovalidate and Lassosum-validate [28]. Some PRS algorithms require hyper-parameter tuning. For P + T and C + T, we used a threshold P of 10^−^^4^ and a pruning r^2^ of 0.2. For the LDpred-grid, we used a fraction of causal variants ρ from a sequence of 10 values from 10^−4^ to 1 on a log-scale, while using the default values for the other hyperparameters (Supplementary Table 1).

Eight candidate polygenic scores were calculated using a validation dataset. To evaluate the performance of the PRS, we computed the Akaike information criterion (AIC) using a linear regression model for SBP, after adjusting for baseline age, sex, standardized PRS_SBP_, and the first 10 principal components (PCs). We selected the best PRS with the lowest AIC. Finally, the LDpred-grid method was used to calculate the PRS for each individual in the test set.

### Statistical analysis

The baseline characteristics of the study population are presented as descriptive statistics for each group of PRS_SBP_ or primary outcomes. A two-sample *t*-test was performed based on the homogeneity of variance for continuous variables. For categorical variables, Pearson’s chi-square test or Fisher’s exact test was used, depending on whether cells with an expected frequency of <5 exceeded 20%.

The association of incident hypertension with genetic factors and sodium intake was analyzed by logistic regression. The model of incident hypertension with a standardized PRS included PRS_SBP_, PRS_DBP_, age, sex, BMI, smoking status, physical activity, sodium intake, potassium intake, interaction of sodium intake and PRS_SBP_ (or interaction of sodium and potassium intake), and the first 10 PCs. In research related to PRS, PRS is categorized into percentiles for easy interpretation, and the results are shown as tertials, quartiles, quintiles, deciles, or the top 10% is compared with the remaining middle and bottom 10% [29, 30]. Although the definition of high PRS has not been established, we use the method of dividing the top 10, middle (10–90 percentiles), and bottom 10 percentiles to compare high risk, average, and low-risk PRS groups, respectively, as in previous studies [31–33]. Kaplan–Meier (K-M) survival curves were obtained for incident hypertension stratified by PRS_SBP_ groups, and the curves between groups were compared using the log-rank test. Statistical analyses were conducted using R (version 3.6.3; R Foundation for Statistical Computing, Vienna, Austria) and the Rex [14].

### Subgroup analyses

Subgroup analyses were performed to examine the effects of sodium intake (or potassium intake) on incident hypertension in each subgroup and to determine their respective influences. The criteria for each subgroup were as follows: sodium/potassium group (quartile 1, quartile 2, quartile 3, or quartile 4), baseline age (<55 or ≥55 years), sex (male or female), BMI (<18.5, 18.5–24.9, or ≥25), smoking (never, ex-smoker, or current), alcohol consumption (no or yes), DM (no or yes), physical activity (no or yes), and PRS group (bottom decile, middle decile 2–9, or top decile). In the subgroup analysis of incident hypertension and sodium intake (or potassium intake), each model was adjusted for baseline age and sex. However, for the subgroup analysis of sodium intake (or potassium intake) with the PRS group, baseline age, sex, and the first 10 PCs were adjusted for in all models.

### Ethical considerations

The Institutional Review Board of Seoul National University Boramae Medical Center approved the study protocol (IRB No. 07-2022-38). This study was conducted in compliance with the Declaration of Helsinki and was conducted using bioresources from the National Biobank of Korea and Korea Disease Control and Prevention Agency, Republic of Korea (NBK-2022-079).

## Results

### Baseline characteristics

Table 1 shows baseline characteristics of participants in the test set. The highest PRS_SBP_ group (top 10%) had significantly higher BMI, baseline blood pressure, red meat intake, and alcohol consumption than the other two groups (bottom 10%, middle), but was the youngest and had the lowest proportion of men. No significant differences were found between the three groups in waist-to-hip ratio (WHR), serum fasting glucose, total cholesterol, low-density lipoprotein (LDL) cholesterol, smoking status, total energy intake, sodium intake, potassium intake, and CVD prevalence. In addition, we also observed the baseline characteristics according to the presence or absence of incident hypertension (Supplementary Table 2). In the test set, in comparison with the non-hypertensive group, the hypertensive group included significantly higher proportions of men, current smokers, patients with DM and CVD, and patients with excessive alcohol consumption. Patients in the hypertensive group also showed lower levels of physical activity. Age, BMI, WHR, SBP, DBP, serum glucose level, low-density lipoprotein cholesterol level, and daily sodium intake were significantly higher in the hypertensive group than in the non-hypertensive group. However, the daily potassium intake was lower in the hypertensive group. The two groups showed no significant differences in terms of total energy intake and daily intake of red meat. Baseline characteristics according to sodium intake and potassium intake are summarized in Supplementary Table 3 and 4.

**Table 1:** Baseline characteristics according to the PRS_SBP_ group

Variable	Total (*N* = 41,351)	Bottom 10% (*N* = 4,136)	Middle (*N* = 33,080)	Top 10% (*N* = 4,135)	*P*
Age (year)	53.0 ± 8.1	53.7 ± 8.1	53.0 ± 8.0	52.3 ± 8.0	<0.001
Male, %	13097 (31.7)	1359 (32.9)	10485 (31.7)	1253 (30.3)	0.043
BMI (kg/m^2^)	23.5 ± 2.7	23.4 ± 2.7	23.5 ± 2.7	23.6 ± 2.8	0.005
WHR	0.9 ± 0.1	0.9 ± 0.1	0.9 ± 0.1	0.9 ± 0.1	0.408
Systolic blood pressure (mmHg)	117.1 ± 11.4	115.1 ± 11.5	117.1 ± 11.4	118.8 ± 11.1	<0.001
Diastolic blood pressure (mmHg)	73.0 ± 7.9	71.9 ± 7.9	73.0 ± 7.8	73.9 ± 7.8	<0.001
Fasting glucose (mg/dL)	93.1 ± 17.5	92.6 ± 15.9	93.1 ± 17.7	92.8 ± 17.1	0.165
Total Cholesterol (mg/dL)	196.9 ± 35.0	197.0 ± 34.6	196.9 ± 34.9	196.6 ± 35.6	0.814
LDL cholesterol (mmol/L)	120.2 ± 31.4	120.5 ± 31.4	120.3 ± 31.3	119.9 ± 31.9	0.669
Smoking status, %					0.091
Never smoker	30964 (75.1)	3073 (74.6)	24776 (75.1)	3115 (75.6)	
Ex-smoker	5667 (13.8)	611 (14.8)	4526 (13.7)	530 (12.9)	
Current smoker	4583 (11.1)	435 (10.6)	3672 (11.1)	476 (11.6)	
Alcohol drinking status, %					<0.001
No	23427 (56.9)	2450 (59.5)	18776 (57.0)	2201 (53.4)	
Yes	17761 (43.1)	1668 (40.5)	14173 (43.0)	1920 (46.6)	
Physical activity, %	21609 (52.4)	2115 (51.3)	17337 (52.6)	2157 (52.4)	0.292
Red meat intake (g/day)	33.6 ± 45.8	31.8 ± 36.2	33.7 ± 45.8	34.9 ± 54.0	0.002
Total energy intake (kcal)	1749.0 ± 555.7	1743.9 ± 540.9	1749.6 ± 556.0	1748.8 ± 568.3	0.825
Potassium intake (g/day)	2.3 ± 1.1	2.2 ± 1.0	2.3 ± 1.1	2.2 ± 1.4	0.629
Sodium intake (g/day)	2.5 ± 1.4	2.5 ± 1.4	2.5 ± 1.4	2.5 ± 1.4	0.645
Diabetes mellitus, %	2766 (6.7)	239 (5.8)	2265 (6.9)	262 (6.3)	0.022
Cardiovascular disease, %	1166 (2.8)	102 (2.5)	952 (2.9)	112 (2.7)	0.287

*BMI* body mass index, *LDL* low-density lipoprotein, *PRS* polygenic risk score, *SBP* systolic blood pressure, *WHR* waist-to-hip ratio

### PRS_SBP,_ PRS_DBP_, and incident hypertension risk

**Fig. 2:**
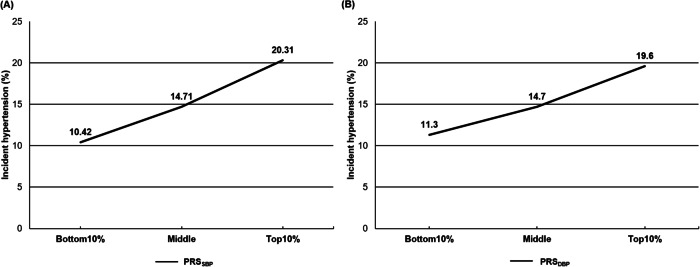
Incidence rate of hypertension according to PRS_SBP,_ PRS_DBP_ groups. The incidence of hypertension gradually increases in the bottom 10%, middle, and top 10% groups of PRS_SBP_ (**A**), and PRS_DBP_ (**B**)

During the 4.5 ± 1.8 years of follow-up, 6137 individuals (14.8%) developed hypertension, and we evaluated the association of PRS_SBP_ with incident hypertension. Most patients were followed up only from baseline to the first health examination, especially the HEXA cohort. The follow-up period for each patient also varied from 1 to 10 years (Supplementary Table 5). We observed a gradual increase in the incidence of hypertension as PRS_SBP_ and PRS_DBP_ levels increased (Fig. 2). Furthermore, the K-M curve demonstrated that the top 10% of individuals with PRS_SBP_ had a significantly higher risk of developing hypertension than those in the other groups (Supplementary Fig. 1). In the logistic regression analyses (Table 2), compared to the middle PRS_SBP_ group (reference), the bottom 10% group had a significantly lower risk of developing hypertension (odds ratio [OR], 0.74; 95% confidence interval [CI], 0.62–0.88), and the top 10% group had an increased risk of developing hypertension (OR, 1.19; 95% CI, 1.03–1.37). In addition, PRS_DBP_ is also an independent risk factor for the development of hypertension, similar to PRS_SBP_.

**Table 2:** Odds ratio for incident hypertension according to PRS, sodium intake, and their interaction

Variables	PRS (continuous variable)		PRS (categorial variable)	
	OR (95% CI)	*P*	OR (95% CI)	*P*
PRS_SBP_ (units of SD)	1.20 (1.14–1.26)	<0.001		
PRS_SBP_ (ref. Middle)				<0.001
Bottom 10%			0.74 (0.62–0.88)	<0.001
Top 10%			1.19 (1.03–1.37)	0.019
PRS_DBP_ (units of SD)	1.05 (1.01–1.09)	0.022		
PRS_DBP_ (ref. Middle)				<0.001
Bottom 10%			0.84 (0.75–0.95)	0.002
Top 10%			1.27 (1.15–1.39)	<0.001
Age (year)	1.04 (1.04–1.04)	<0.001	1.04 (1.03–1.04)	<0.001
Sex, female (ref. male)	0.78 (0.71–0.85)	<0.001	0.78 (0.72–0.85)	<0.001
BMI (kg/m^2^)	1.15 (1.14–1.16)	<0.001	1.15 (1.14–1.16)	<0.001
Smoking status (ref. Never smoker)		0.022		0.019
Ex-smoker	1.02 (0.92–1.13)	0.707	1.02 (0.92–1.13)	0.680
Current smoker	1.15 (1.14–1.16)	0.010	1.15 (1.04–1.28)	0.009
Physical activity (ref. No)	0.89 (0.84–0.94)	<0.001	0.89 (0.84–0.94)	<0.001
Sodium intake ≥ 2.0 g/day (ref. <2.0 g/day)	1.10 (1.03–1.18)	0.003	1.08 (1.00-1.16)	<0.001
Potassium intake (g/day)	0.96 (0.93–0.99)	0.015	0.96 (0.93–0.99)	0.014
Interaction: sodium intake ≥ 2.0 g/day, PRS_SBP_ (ref. Sodium <2.0 g/day, PRS_SBP_)		0.097		
Sodium ≥ 2.0 g/day, PRS_SBP_	1.05 (0.99–1.11)	0.096		
Interaction: sodium intake, PRS_**SBP**_ (ref. Sodium <2.0 g/day, Middle)				0.015
Sodium intake ≥ 2.0 g/day, Bottom 10%			0.92 (0.74–1.14)	0.439
Sodium intake ≥ 2.0 g/day, Top 10%			1.27 (1.07–1.51)	0.007

*BMI* body mass index, *CI* confidence interval, *DBP* diastolic blood pressure, *OR* odds ratio, *PRS* polygenic risk score, *SBP* systolic blood pressure, *SD* standard deviation

Multivariable logistic regression, adjusted for PRS_SBP_, PRS_DBP_, age, sex, BMI, smoking status, physical activity, sodium intake, potassium intake, interaction of sodium intake and PRS_SBP_, and the first 10 principal components.

### Risk factors for incident hypertension focusing on sodium and potassium intake

We sought to identify the risk factors for incident hypertension, including sodium and potassium intakes, and evaluated their association with PRS_SBP_ and PRS_DBP_. The rate of incident hypertension gradually increased with higher sodium intake (Supplementary Fig. 2A), and even after adjusting for PRS_SBP_ and PRS_DBP_ as well as other risk factors for hypertension, sodium intake was significantly associated with an increased incidence of hypertension (OR, 1.10; 95% CI, 1.03–1.18; *P* = 0.003). There was no statistical significance in the interaction between continuous variable of PRS_SBP_ and sodium intake, but a significant interaction was observed in the sodium intake ≥2.0 g/day, PRS_SBP_ top 10% group compared to the sodium intake <2.0 g/day, PRS_SBP_ middle group (Table 2). Interaction of PRS_DBP_ with sodium intake on incident hypertension, both as a continuous and categorical variable, was not significant (Supplementary Table 6). In addition, sodium intake was significantly associated with incident hypertension in individuals in the middle and top 10% of the PRS_SBP_ groups with ORs of 1.03 (95% CI, 1.01–1.06; *P* = 0.002) and 1.08 (95% CI, 1.02–1.14; *P* = 0.008), respectively (Table 3). This significant association was also found in the middle PRS_DBP_ groups. Although a trend of increased risk of hypertension due to sodium intake was identified in the top 10% PRS_DBP_ group (OR, 1.05; 95% CI, 0.99–1.10; *P* = 0.061), statistical significance was not proved.

**Table 3:** Odds ratio of dietary sodium intake for incident hypertension according to subgroups

Subgroup	*N*	Multivariable	
		OR (95% CI)	*P*
PRS_SBP_			
Bottom 10%	4,136	1.03 (0.96–1.11)	0.412
Middle	33,080	1.03 (1.01–1.06)	0.002
Top 10%	4,135	1.08 (1.02–1.14)	0.008
PRS_DBP_			
Bottom 10%	4,136	1.01 (0.94–1.08)	0.845
Middle	33,080	1.04 (1.02–1.06)	<0.001
Top 10%	4,135	1.05 (0.99–1.10)	0.061
Age			
<55 years old	24,517	1.03 (1.00–1.06)	0.052
≥ 55 years old	16,834	1.04 (1.01–1.07)	0.004
Sex			
Male	13,097	1.03 (1.00–1.06)	0.027
Female	28,254	1.04 (1.01–1.07)	0.002
BMI			
<18.5 kg/m^2^	870	0.93 (0.74–1.18)	0.566
18.5–24.9 kg/m^2^	29,320	1.02 (0.99–1.04)	0.203
≥ 25.0 kg/m^2^	11,155	1.04 (1.01–1.07)	0.006
Smoking status			
Never smoker	30,964	1.04 (1.02–1.07)	<0.001
Ex-smoker	5667	1.04 (0.99–1.09)	0.129
Current smoker	4583	1.00 (0.96–1.05)	0.932
Alcohol drinking			
No	23,427	1.04 (1.01–1.07)	0.005
Yes	17,761	1.03 (1.01–1.06)	0.022
Physical activity			
No	19,601	1.03 (1.00–1.05)	0.082
Yes	21,609	1.05 (1.03–1.08)	<0.001
Diabetes mellitus			
No	38,585	1.04 (1.02–1.06)	<0.001
Yes	2766	1.01 (0.94–1.08)	0.813

*BMI* body mass index, *CI* confidence interval, *DBP* diastolic blood pressure, *OR* odds ratio, *PRS* polygenic risk score, *SBP* systolic blood pressure, *SD* standard deviation

Multivariable logistic regression, adjusted for age, sex, sodium intake (continuous variable, g/day).

In contrast, potassium intake was associated with a lower risk of hypertension (OR, 0.96; 95% CI, 0.93–0.99; *P* = 0.015) (Table 2), and the incident hypertension rate gradually decreased with higher potassium intake (Supplementary Figure 2B). In addition, age (OR, 1.04; 95% CI, 1.04–1.04; *P* < 0.001), female (OR, 0.78; 95% CI, 0.71–0.85; *P* < 0.001), BMI (OR, 1.15; 95% CI, 1.14–1.16; *P* < 0.001), current smoker (OR, 1.15; 95% CI, 1.14–1.16; *P* = 0.010) and physical activity (OR, 0.89; 95% CI, 0.84–0.94; *P* < 0.001) were significantly associated with the incident hypertension (Table 2). There was no statistically significant interaction between sodium and potassium intake (Supplementary Table 7).

### Associations of sodium intake with incident hypertension by subgroup

Table 3 shows the results of the subgroup analysis of the association between incident hypertension and sodium intake. We observed that sodium intake was significantly associated with incident hypertension in individuals aged ≥ 55 years (OR, 1.04; 95% CI, 1.01–1.07), those with BMI ≥ 25 kg/m^2^ (OR, 1.04; 95% CI, 1.01–1.07), never-smokers (OR, 1.04; 95% CI, 1.02–1.07), patients without DM (OR, 1.04; 95% CI, 1.02–1.06; *P* < 0.001), and those who engaged in physical activity (OR, 1.05; 95% CI, 1.03–1.08; *P* < 0.001). These significant associations were consistent across all subgroups stratified according to sex and alcohol intake. Additionally, we investigated the relationship between potassium intake and incident hypertension using subgroup analyses, but no statistically significant relationships were observed (Supplementary Table 8).

## Discussion

Using a large-scale nationwide prospective cohort, we found that the PRS_SBP_, PRS_DBP_, high sodium intake, and low potassium intake were significantly associated with incident hypertension in an Asian population. Sodium intake was an independent risk factor for incident hypertension, its effect on incident hypertension interacts with hereditary factors in the sodium intake ≥2.0 g/day, PRS_SBP_ top 10% group. The elderly, obese, and high PRS_SBP_ groups were at a comparatively greater risk of developing hypertension owing to sodium intake, underscoring the significance of lifestyle and dietary control in preventing hypertension.

A previous study evaluated the PRS_SBP_ in association with uncontrolled hypertension and major adverse cardiovascular events using the UK Biobank (UKB) and including patients of White British ancestry who were diagnosed as having hypertension and took antihypertensive medications [8]. Another study by Vaura et al. revealed that the PRS for blood pressure could predict hypertension risk and earlier hypertension onset in FINRISK participants who were recruited from hospital biobanks or disease-based cohorts, and were mainly of European ancestry [9]. Kurniansyah et al. assessed diverse cohorts and biobanks, analyzed 52,436 individuals, and showed that the PRS for hypertension was associated with both prevalent and incident hypertension. Although these studies included patients of diverse ethnic backgrounds, Asians only constituted a minority in these studies [10]. Parcha et al. showed that PRS_SBP_ is associated with BP traits and CVD using a multi-ancestry Pan UKB GWAS [34]; this study included only 2.8% of Asian patients. Most of these studies were primarily conducted in Western populations and investigated the association between BP PRS and hypertension, treatment responsiveness of hypertension, or the occurrence of CVD. However, none of these studies investigated the relationships of sodium and potassium intake with hypertension.

Only one previous study conducted in an Asian population showed that BP PRS was related to hypertension and BP traits in a Japanese population [11]. However, this study did not evaluate the incidence of hypertension and did not adjust for sodium and potassium intake. In addition, while a previous study examined the relationship between PRS, hypertension, and PRS performance using 3376 test set participants, our report presents a large-scale study of 41,351 test set participants that evaluated the relationship between PRS_SBP_, PRS_DBP_ and incident hypertension.

Previous studies on this topic showed that PRS_SBP_ is significantly associated with hypertension and CVD. However, most of these studies related to PRS_SBP_ were conducted in Western populations. Although one study targeted Asian, evidence for an association between incident hypertension and PRS_SBP_ in Asian populations is scarce. In addition, no study has evaluated other hypertension-related clinical factors, including diet, in relation to PRS_SBP_. In this respect, our study is meaningful in investigating the effects of various factors of clinical importance, including PRS_SBP_, PRS_DBP_ and sodium and potassium intake, on incident hypertension in Asians using a large, nationwide prospective cohort study.

Our study showed that the PRS_SBP_ was significantly associated with incident hypertension, which is consistent with the findings of previous studies [8, 9, 11]. This may have occurred because individuals with a genetic predisposition develop hypertension earlier or tend to have a higher BP. Nevertheless, this finding suggests that the high-risk group for hypertension can be predicted in advance through genetic surveillance. Moreover, in the high-risk group, early detection by regular monitoring of BP and lifestyle modifications to prevent hypertension can improve the management of hypertension.

Dietary sodium intake was significantly associated with the development of hypertension even after adjusting for age, sex, physical activity, BMI, comorbidities, PRS_DBP_, and PRS_SBP_ which are known risk factors for hypertension. Several previous studies have shown an association between sodium intake and hypertension and reported that reducing sodium intake lowers BP [35–39]. The American Heart Association and the U.S. Food and Drug Administration (FDA) recently recommended limiting sodium intake to prevent hypertension and CVD [40, 41]. Sodium is a major component of the extracellular fluid (ECF) and a major determinant of plasma osmolality. An increase in sodium intake leads to water retention to maintain constant serum osmolarity and sodium concentration, resulting in increased kidney perfusion and pressure natriuresis. However, reduced sodium excretion from the kidneys can also lead to hypertension [42, 43]. Other mechanisms by which sodium intake contributes to hypertension include high sodium intake-induced endothelial dysfunction and vascular resistance [44, 45]. Our study provides additional clinical evidence supporting these findings. In particular, dietary sodium intake was significantly associated with incident hypertension in participants in the PRS_SBP_ middle and top 10% groups (i.e., participants with a high genetic risk of hypertension). Although the findings showed statistically significant interaction between PRS_SBP_ and sodium intake for incident hypertension only in the PRS_SBP_ categorical group: sodium intake ≥2.0 g/day, PRS_SBP_ top 10% group, this is the first study to evaluate the relationship between PRS_SBP_ and sodium intake and their effects on the development of hypertension. We suggest that dietary sodium restriction is important for everyone but is especially emphasized in the moderate or high PRS_SBP_ group.

Our study also showed that potassium intake was inversely related to hypertension. This finding is also consistent with the results of previous studies [37, 46–49], except for one meta-analysis that showed a J-shaped relationship between potassium intake and hypertension [50]. Although our study focused on sodium intake and PRS_SBP_, potassium intake is also an important factor in the development of hypertension. Thus, our results suggested that the risk of incident hypertension increased in the group with higher PRS_SBP_, higher sodium intake, and lower potassium intake. The findings also indicated that dietary sodium restriction may be more important for preventing hypertension in groups with higher PRS_SBP,_ older age, female sex, and high BMI.

Our study had several limitations. First, for our primary analysis, we used logistic rather than Cox regression analysis. Participants in this study were not followed up regularly, and the interval of the first follow-up from the baseline survey varied widely from 1 to 10 years. Classifying the participant as having incident hypertension did not occur at the exact time of incident hypertension. There is a time interval of months to years between the hypertension detection at the survey and the actual incident hypertension, depending on the individual. Therefore, we used logistic rather than Cox analysis, which is based on the accuracy of time to event. Second, the KoGES is a community population-based prospective cohort that collected participants aged 40 or older and is not a representative cohort for Korean as a whole. Additionally, because we had assessed the risk of incident hypertension though a retrospective analysis, we had to exclude individuals who had hypertension at baseline. Therefore, there are concerns about collider-stratification bias. However, the odds ratio estimation in the logistic regression model is known to be unaffected by sampling bias [51]. Our results were limited to Asian populations; therefore, they cannot be generalized to other ethnicities. Nevertheless, these findings are meaningful considering the lack of previous studies on the Asian population. Third, this study did not adjust for a family history of hypertension. However, assessments of a family history of hypertension may not be very accurate when performed with a simple questionnaire. Fourth, we focused only on sodium and potassium intake in the diet and did not investigate other diets with vegetables and nuts, such as the Dietary Approaches to Stop Hypertension (DASH) diet. Moreover, the use of questionnaires to assess sodium and potassium intake may have reduced the accuracy of the data collected. Nevertheless, this is the first study to use PRS analysis to show that dietary sodium and potassium have significant effects on incident hypertension even after adjusting for genetic factors, and the results can serve as additional evidence for explaining the importance of low-sodium, high-potassium diet for hypertension prevention. Finally, due to the characteristics of an observational cohort study, we could not conclude that frequent BP monitoring is needed in the group with a higher PRS_SBP_ or that hypertension was prevented by low-sodium, high-potassium diet. However, our study supports the need for a genetic approach and dietary efforts to prevent hypertension, and further studies on individualized approaches and prevention are needed.

### Perspective of Asia

The risk of hypertension varies among different ethnicities. The impact of sodium intake on hypertension development may also differ due to genetic factors [52]. The significant interaction between genetic factors and a high-salt diet, as well as the elevated risk of hypertension associated with genetic factors, may vary in other Asian countries and ethnic groups [53]. Therefore, we warrant additional research on the genetic risk and its interaction with dietary sodium intake for incident hypertension.

## Conclusions

We developed PRS_SBP_, and PRS_DBP_ for Asians, including Koreans, and demonstrated that PRS_SBP_, and sodium, potassium intake were independent risk factors for incident hypertension. Dietary sodium restriction is necessary to prevent hypertension, and its importance is especially prominent in patients at a high risk of hypertension due to sodium intake.

## Supplementary information

Supplementary Figures

Supplementary Tables

## Data Availability

Data will be released on a limited basis by the corresponding author upon request.
