# Correction: Effects of polygenic risk score and sodium and potassium intake on hypertension in Asians: A nationwide prospective cohort study

**DOI:** 10.1038/s41440-024-01912-3

**Published:** 2024-09-25

**Authors:** Eunjin Bae, Yunmi Ji, Jinyeon Jo, Yaerim Kim, Jung Pyo Lee, Sungho Won, Jeonghwan Lee

**Affiliations:** 1 Department of Internal Medicine, Gyeongsang National University Changwon Hospital, Changwon, Republic of Korea; 2 Department of Internal Medicine, College of Medicine, Gyeongsang National University, Jinju, Republic of Korea; 3 Institute of Medical Science, College of Medicine, Gyeongsang National University, Jinju, Republic of Korea; 4 College of Natural Sciences, Interdisciplinary Program in Bioinformatics, Seoul National University, Seoul, Republic of Korea; 5 Department of Public Health Sciences, Institute of Health & Environment, Seoul National University, Seoul, Republic of Korea; 6 Department of Internal Medicine, College of Medicine, Keimyung University School of Medicine, Daegu, Republic of Korea; 7 Department of Internal Medicine, Seoul National University Boramae Medical Center, Seoul, Republic of Korea; 8 Department of Internal Medicine, Seoul National University College of Medicine, Seoul, Republic of Korea; 9 Interdisciplinary Program for Bioinformatics, College of Natural Science, Seoul National University, Seoul, Republic of Korea; 10 RexSoft Corps, Seoul, Republic of Korea

Correction to: *Hypertension Research*

10.1038/s41440-024-01784-7

published online 10 July 2024

In the original version of this article, the given and family names of Eunjin Bae were incorrectly structured. The name was displayed correctly in all versions at the time of publication.

The original article has been corrected.

