# Supplementary material for: Effects of polygenic risk score and sodium and potassium intake on hypertension in Asians: A nationwide prospective cohort study
Source: Hypertens Res. 2024 Jul 10;47(11):3045–55. doi: 10.1038/s41440-024-01784-7 (PMC11534693; doi:10.1038/s41440-024-01784-7)
Supplement: Supplementary file 1 — Supplementary Figures [file 41440_2024_1784_MOESM1_ESM.pptx]

## Slide 1
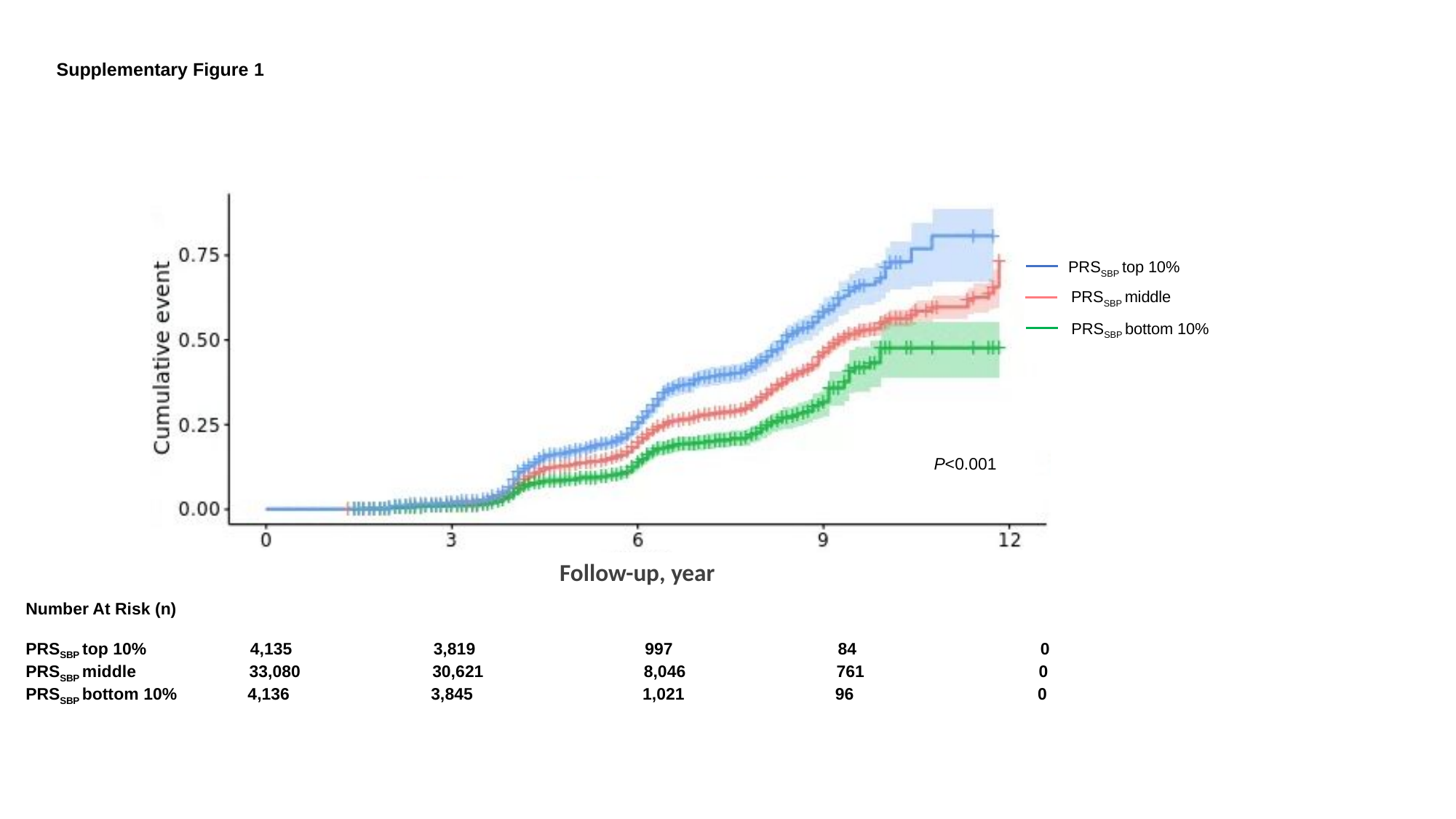

Supplementary Figure 1
PRSSBP top 10%
PRSSBP middle
PRSSBP bottom 10%
P<0.001
Follow-up, year
Number At Risk (n)
PRSSBP top 10% 4,135 3,819 997 84 0
PRSSBP middle 33,080 30,621 8,046 761 0
PRSSBP bottom 10% 4,136 3,845 1,021 96 0

## Slide 2
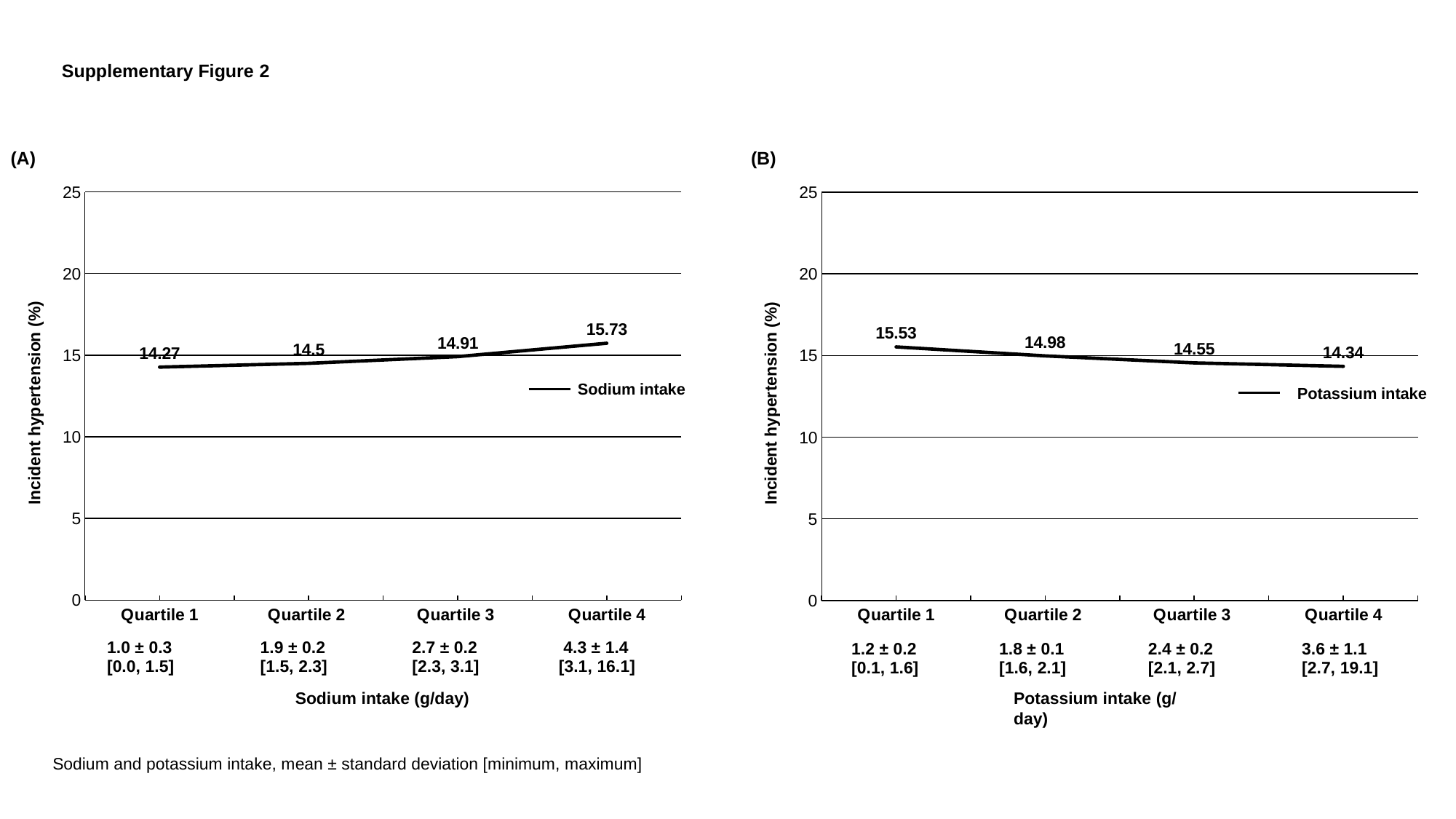

Supplementary Figure 2
(A)
(B)
### Chart
| Category | Dietary sodium intake |
|---|---|
| Quartile 1 | 14.27 |
| Quartile 2 | 14.5 |
| Quartile 3 | 14.91 |
| Quartile 4 | 15.73 |
### Chart
| Category | Dietary potassium intake |
|---|---|
| Quartile 1 | 15.53 |
| Quartile 2 | 14.98 |
| Quartile 3 | 14.55 |
| Quartile 4 | 14.34 |Sodium intake
Potassium intake
| 1.0 ± 0.3 [0.0, 1.5] | 1.9 ± 0.2 [1.5, 2.3] | 2.7 ± 0.2 [2.3, 3.1] | 4.3 ± 1.4 [3.1, 16.1] |
| --- | --- | --- | --- |
| 1.2 ± 0.2 [0.1, 1.6] | 1.8 ± 0.1 [1.6, 2.1] | 2.4 ± 0.2 [2.1, 2.7] | 3.6 ± 1.1 [2.7, 19.1] |
| --- | --- | --- | --- |
Sodium intake (g/day)
Potassium intake (g/day)
Sodium and potassium intake, mean ± standard deviation [minimum, maximum]
