# Supplementary material for: Effects of polygenic risk score and sodium and potassium intake on hypertension in Asians: A nationwide prospective cohort study
Source: Hypertens Res. 2024 Jul 10;47(11):3045–55. doi: 10.1038/s41440-024-01784-7 (PMC11534693; doi:10.1038/s41440-024-01784-7)
Supplement: Supplementary file 2 — Supplementary Tables [file 41440_2024_1784_MOESM2_ESM.docx]

**Supplementary Table 1. Validation set PRS summary**

| **MAF** | **Mtehod** | **Correaltion** | **AIC** | **BETA** | **Statistics** | ***P*-value** |
| --- | --- | --- | --- | --- | --- | --- |
| JPB0 | P+T | 0.0818 | 46061.58 | 1.5051 | 6.667 | 2.88E-11 |
| JPB0 | C+T | 0.1149 | 46023.69 | 2.0444 | 9.093 | 1.33E-19 |
| JPB0 | PRScs | 0.1296 | 45991.37 | 2.4094 | 10.748 | 1.13E-26 |
| JPB0 | LDpred-inf | 0.1169 | 46012.35 | 2.1802 | 9.705 | 4.35E-22 |
| JPB0 | LDpred-auto | 0.1484 | 45957.42 | 2.7379 | 12.256 | 4.41E-34 |
| JPB0 | LDpred-grid | 0.1488 | 45958.11 | 2.7313 | 12.227 | 6.22E-34 |
| JPB0 | LASSOsum-v | -0.0202 | 46105.95 | -0.0280 | -0.124 | 9.02E-01 |
| JPB0 | LASSOsum-pv | 0.1455 | 45971.07 | 2.6131 | 11.671 | 4.19E-31 |
| JPB0.005 | P+T | 0.0818 | 46061.58 | 1.5051 | 6.667 | 2.88E-11 |
| JPB0.005 | C+T | 0.1149 | 46023.69 | 2.0444 | 9.093 | 1.33E-19 |
| JPB0.005 | PRScs | 0.1275 | 45996.19 | 2.3582 | 10.517 | 1.27E-25 |
| JPB0.005 | LDpred-inf | 0.1165 | 46012.73 | 2.1755 | 9.685 | 5.27E-22 |
| JPB0.005 | LDpred-auto | 0.1481 | 45958.13 | 2.7316 | 12.226 | 6.30E-34 |
| JPB0.005 | LDpred-grid | 0.1476 | 45958.27 | 2.7303 | 12.220 | 6.76E-34 |
| JPB0.005 | LASSOsum-v | -0.0199 | 46105.95 | -0.0244 | -0.107 | 9.15E-01 |
| JPB0.005 | LASSOsum-pv | 0.1455 | 45971.12 | 2.6127 | 11.669 | 4.28E-31 |
| JPB0.01 | P+T | 0.0818 | 46061.58 | 1.5051 | 6.667 | 2.88E-11 |
| JPB0.01 | C+T | 0.1154 | 46022.59 | 2.0579 | 9.154 | 7.62E-20 |
| JPB0.01 | PRScs | 0.1303 | 45991.63 | 2.4062 | 10.735 | 1.29E-26 |
| JPB0.01 | LDpred-inf | 0.1173 | 46012.12 | 2.1826 | 9.716 | 3.88E-22 |
| JPB0.01 | LDpred-auto | 0.1483 | 45957.99 | 2.7330 | 12.232 | 5.88E-34 |
| JPB0.01 | LDpred-grid | 0.1491 | 45955.55 | 2.7551 | 12.334 | 1.72E-34 |
| JPB0.01 | LASSOsum-v | -0.0199 | 46105.95 | -0.0242 | -0.107 | 9.15E-01 |
| JPB0.01 | LASSOsum-pv | 0.1388 | 45980.47 | 2.5229 | 11.253 | 4.70E-29 |
| JPB0.05 | P+T | 0.0800 | 46063.2 | 1.4775 | 6.544 | 6.55E-11 |
| JPB0.05 | C+T | 0.1161 | 46021.12 | 2.0758 | 9.235 | 3.62E-20 |
| JPB0.05 | PRScs | 0.1311 | 45989.88 | 2.4249 | 10.818 | 5.33E-27 |
| JPB0.05 | LDpred-inf | 0.1185 | 46010.23 | 2.2047 | 9.815 | 1.50E-22 |
| JPB0.05 | LDpred-auto | 0.1496 | 45955.93 | 2.7521 | 12.318 | 2.09E-34 |
| JPB0.05 | LDpred-grid | 0.1497 | 45955.88 | 2.7524 | 12.320 | 2.03E-34 |
| JPB0.05 | LASSOsum-v | -0.0193 | 46105.96 | -0.0118 | -0.052 | 9.58E-01 |
| JPB0.05 | LASSOsum-pv | 0.1371 | 45982.08 | 2.5064 | 11.179 | 1.06E-28 |

**Supplementary Table 2. Baseline characteristics according to the incident hypertension and normal blood pressure**

| Variable | Validation Set | | | Test set | | |
| --- | --- | --- | --- | --- | --- | --- |
|  | **Normal blood pressure**  **(N=2,205)** | **Incident hypertension**  **(N=1,552)** | ***P*-value** | **Normal blood pressure**  **(N=35,214)** | **Incident hypertension**  **(N=6,137)** | ***P*-value** |
| Age (year) | 48.4 ± 7.4 | 52.5 ± 8.6 | <0.001 | 52.6 ± 8.0 | 55.2 ± 8.2 | <0.001 |
| Male, % | 1006 (45.6) | 760 (49.0) | 0.047 | 10639 (30.2) | 2458 (40.1) | <0.001 |
| BMI (kg/m^2^) | 23.78 (2.79) | 24.74 (3.00) | <0.001 | 23.3 ± 2.7 | 24.5 ± 2.8 | <0.001 |
| WHR | 0.85 ± 0.07 | 0.88 ± 0.06 | <0.001 | 0.85 ± 0.07 | 0.89 ± 0.07 | <0.001 |
| Systolic blood pressure (mmHg) | 108.3 ± 10.4 | 118.66 ± 10.0 | <0.001 | 115.8 ± 11.2 | 124.7 ± 9.3 | <0.001 |
| Diastolic blood pressure (mmHg) | 72.7 ± 7.7 | 78.6 ± 6.8 | <0.001 | 72.2 ± 7.8 | 77.7 ± 6.6 | <0.001 |
| Fasting glucose (mg/dL) | 85.0 ± 17.1 | 87.0 ± 20.8 | 0.001 | 92.5 ± 17.0 | 96.0 ± 20.1 | <0.001 |
| Total Cholesterol (mg/dL) | 188.4 ± 33.7 | 190.8 ± 34.6 | 0.038 | 196.4 ± 34.8 | 199.7 ± 36.0 | <0.001 |
| LDL cholesterol (mmol/L) | 114.9 ± 30.4 | 114.8 ± 31.6 | 0.872 | 119.8 ± 31.1 | 122.7 ± 32.5 | <0.001 |
| Smoking status, % |  |  | <0.001 |  |  | 0.003 |
| Never smoker | 26755 (76.2) | 4209 (68.8) | <0.001 | 1347 (61.7) | 877 (57.4) |  |
| Ex-smoker | 4606 (13.1) | 1061 (17.3) | <0.001 | 340 (15.6) | 229 (15.0) |  |
| Current smoker | 3733 (10.6) | 850 (13.9) | <0.001 | 496 (22.7) | 421 (27.6) |  |
| Alcohol drinker status, % |  |  | <0.001 |  |  | 0.272 |
| No | 20151 (57.5) | 3276 (53.5) |  | 1129 (51.6) | 766 (49.7) |  |
| Yes | 14916 (42.5) | 2845 (46.5) |  | 1058 (48.4) | 774 (50.3) |  |
| Physical activity, % | 18540 (52.8) | 3069 (50.2) | <0.001 | 1188 (54.9) | 956 (63.0) | <0.001 |
| Daily red meat intake (g) | 46.0 ± 50.9 | 43.5 ± 53.7 | 0.151 | 33.8 ± 45.0 | 32.6 ± 50.0 | 0.064 |
| Total energy intake (kcal) | 1956.4 ± 660.6 | 1977.2 ± 694.2 | 0.364 | 1750.4 ± 558.2 | 1740.7 ± 541.4 | 0.197 |
| Potassium intake (g) | 2.57 ± 1.2 | 2.6 ± 1.2 | 0.973 | 2.3 ± 1.1 | 2.2 ± 1.1 | 0.035 |
| Sodium intake (g) | 3.12 ± 1.5 | 3.2 ± 1.7 | 0.041 | 2.5 ± 1.4 | 2.6 ± 1.5 | <0.001 |
| Diabetes mellitus, % | 152 (6.9) | 180 (11.6) | <0.001 | 2227 (6.3) | 539 (8.8) | <0.001 |
| Cardiovascular disease, % | 24 (1.1) | 30 (1.9) | 0.044 | 962 (2.7) | 204 (3.3) | 0.011 |

BMI, body mass index; LDL, low-density lipoprotein; PRS, polygenic risk score; SBP, systolic blood pressure; WHR, waist-to-hip ratio

**Supplementary Table 3. Baseline characteristics according to the sodium intake group**

| Variable | Total  (N=41,351) | Sodium <2.0 g/d  (N=16,936) | Sodium ≥2.0 g/d  (N=24,097) | *P* |
| --- | --- | --- | --- | --- |
| Age (year) | 53.0 ± 8.1 | 53.2 ± 8.0 | 52.7 ± 8.1 | <0.001 |
| Male, % | 13097 (31.7) | 4689 (27.7) | 8328 (34.6) | <0.001 |
| BMI (kg/m^2^) | 23.5 ± 2.7 | 23.3 ± 2.7 | 23.6 ± 2.8 | <0.001 |
| WHR | 0.9 ± 0.1 | 0.9 ± 0.1 | 0.9 ± 0.1 | <0.001 |
| Systolic blood pressure (mmHg) | 117.1 ± 11.4 | 116.8 ± 11.5 | 117.3 ± 11.3 | <0.001 |
| Diastolic blood pressure (mmHg) | 73.0 ± 7.9 | 72.69 ± 7.9 | 73.2 ± 7.8 | <0.001 |
| Fasting glucose (mg/dL) | 93.1 ± 17.5 | 92.8 ± 17.4 | 93.2 ± 17.6 | 0.029 |
| Total Cholesterol (mg/dL) | 196.9 ± 35.0 | 196.87 ± 35.0 | 196.9 ± 34.9 | 0.935 |
| LDL cholesterol (mmol/L) | 120.2 ± 31.4 | 120.4 ± 31.3 | 120.2 ± 31.4 | 0.547 |
| Smoking status, % |  |  |  | <0.001 |
| Never smoker | 30964 (75.1) | 13200 (78.1) | 17552 (73.0) |  |
| Ex-smoker | 5667 (13.8) | 2110 (12.5) | 3526 (14.7) |  |
| Current smoker | 4583 (11.1) | 1592 (9.4) | 2969 (12.4) |  |
| Alcohol drinker status, % |  |  |  | <0.001 |
| No | 23427 (56.9) | 10068 (59.6) | 13207 (55.0) |  |
| Yes | 17761 (43.1) | 6825 (40.4) | 10825 (45.0) |  |
| Physical activity, % | 21609 (52.4) | 8696 (51.5) | 12787 (53.2) | 0.001 |
| Red meat intake (g/day) | 33.6 ± 45.8 | 25.31 ± 28.8 | 39.47 ± 53.9 | <0.001 |
| Total energy intake (kcal) | 1749.0 ± 555.7 | 1501.6 ± 393.8 | 1922.8 ± 586.2 | <0.001 |
| Potassium intake (g/day) | 2.3 ± 1.1 | 1.59 ± 0.6 | 2.70 ± 1.1 | <0.001 |
| Diabetes mellitus, % | 2766 (6.7) | 1109 (6.6) | 1634 (6.8) | 0.363 |
| Cardiovascular disease, % | 1166 (2.8) | 491 (2.9) | 664 (2.8) | 0.403 |

BMI, body mass index; LDL, low-density lipoprotein; PRS, polygenic risk score; SBP, systolic blood pressure; WHR, waist-to-hip ratio

**Supplementary Table 4. Baseline characteristics according to the potassium intake group**

| Variable | Total  (N=41,351) | Potassium <3.5 g/d  (N=37,100) | Potassium ≥3.5 g/d  (N=3,933) | *P* |
| --- | --- | --- | --- | --- |
| Age (year) | 53.0 ± 8.1 | 53.1 ± 8.1 | 51.9 ± 7.6 | <0.001 |
| Male, % | 13097 (31.7) | 11804 (31.8) | 1213 (30.8) | 0.218 |
| BMI (kg/m^2^) | 23.5 ± 2.7 | 23.5 ± 2.7 | 23.7 ± 2.7 | <0.001 |
| WHR | 0.9 ± 0.1 | 0.9 ± 0.1 | 0.9 ± 0.1 | 0.002 |
| Systolic blood pressure (mmHg) | 117.1 ± 11.4 | 117.1 ± 11.4 | 116.7 ± 11.4 | 0.021 |
| Diastolic blood pressure (mmHg) | 73.0 ± 7.9 | 73.0 ± 7.9 | 73.2 ± 7.8 | 0.076 |
| Fasting glucose (mg/dL) | 93.1 ± 17.5 | 93.2 ± 17.6 | 92.4 ± 16.7 | 0.008 |
| Total Cholesterol (mg/dL) | 196.9 ± 35.0 | 196.8 ± 34.9 | 197.7 ± 35.2 | 0.149 |
| LDL cholesterol (mmol/L) | 120.2 ± 31.4 | 120.2 ± 31.3 | 120.7 ± 31.6 | 0.36 |
| Smoking status, % |  |  |  | 0.003 |
| Never smoker | 30964 (75.1) | 27770 (75.0) | 2982 (76.1) |  |
| Ex-smoker | 5667 (13.8) | 5163 (13.9) | 473 (12.1) |  |
| Current smoker | 4583 (11.1) | 4098 (11.1) | 463 (11.8) |  |
| Alcohol drinker status, % |  |  |  | 0.082 |
| No | 23427 (56.9) | 21099 (57.0) | 2176 (55.6) |  |
| Yes | 17761 (43.1) | 15909 (43.0) | 1741 (44.5) |  |
| Physical activity, % | 21609 (52.4) | 19083 (51.5) | 2400 (61.2) | <0.001 |
| Red meat intake (g/day) | 33.6 ± 45.8 | 29.8 ± 31.5 | 69.6 ± 105.5 | <0.001 |
| Total energy intake (kcal) | 1749.0 ± 555.7 | 1657.9 ± 436.1 | 2608.1 ± 782.3 | <0.001 |
| Sodium intake (g/day) | 2.5 ± 1.4 | 2.23 ± 1.1 | 4.8 ± 2.0 | <0.001 |
| Diabetes mellitus, % | 2766 (6.7) | 2509 (6.8) | 234 (6.0) | 0.056 |
| Cardiovascular disease, % | 1166 (2.8) | 1062 (2.9) | 93 (2.4) | 0.083 |

BMI, body mass index; LDL, low-density lipoprotein; PRS, polygenic risk score; SBP, systolic blood pressure; WHR, waist-to-hip ratio

**Supplementary Table 5. Follow-up period and number of observations for each cohort**

| Cohort | Period | Baseline – 1^st^ | 1^st^ – 2^nd^ | 2^nd^ – 3^rd^ | 3^rd^ – 4^th^ |
| --- | --- | --- | --- | --- | --- |
| CAVAS  (N = 4,539) | 1 year | 760 | 154 | 39 | 1 |
|  | 2 years | 2238 | 801 | 295 | 40 |
|  | 3 years | 673 | 1568 | 693 | 75 |
|  | 4 years | 312 | 564 | 407 | 1 |
|  | 5 years | 312 | 173 | 65 |  |
|  | 6 years | 115 | 120 | 20 |  |
|  | 7 years | 42 | 79 |  |  |
|  | 8 years | 41 | 17 |  |  |
|  | 9 years | 37 | 6 |  |  |
|  | 10 years | 4 |  |  |  |
|  | 11 years | 5 |  |  |  |
| HEXA  (N = 36,812) | 1 year | 789 |  |  |  |
|  | 2 years | 1790 |  |  |  |
|  | 3 years | 10329 |  |  |  |
|  | 4 years | 12932 |  |  |  |
|  | 5 years | 3325 |  |  |  |
|  | 6 years | 4637 |  |  |  |
|  | 7 years | 1057 |  |  |  |
|  | 8 years | 1563 |  |  |  |
|  | 9 years | 351 |  |  |  |
|  | 10 years | 39 |  |  |  |

CAVAS, cardiovascular disease association study; HEXA, health examination study

**Supplementary Table 6. Odds ratio for incident hypertension according to PRS, sodium intake, and their interaction (PRS_DBP_ and sodium intake)**

| **Variables** | **PRS (continuous variable)** | | **PRS (categorial variable)** | |
| --- | --- | --- | --- | --- |
|  | **OR (95% CI)** | **P** | **OR (95% CI)** | **P** |
| PRS_SBP_ (units of SD) | 1.23 (1.18–1.28) | <0.001 |  |  |
| PRS**_SBP_** (ref. Middle) |  |  |  | <0.001 |
| Bottom 10% |  |  | 0.70 (0.63–0.79) | <0.001 |
| Top 10% |  |  | 1.37 (1.25–1.51) | 0.019 |
| PRS**_DBP_** (units of SD) | 1.02 (0.97–1.08) | 0.374 |  |  |
| PRS**_DBP_** (ref. Middle) |  |  |  | <0.001 |
| Bottom 10% |  |  | 0.91 (0.77–1.07) | 0.259 |
| Top 10% |  |  | 1.22 (1.05–1.40) | 0.008 |
| Age (year) | 1.04 (1.04–1.04) | <0.001 | 1.04 (1.03–1.04) | <0.001 |
| Sex, female (ref. male) | 0.78 (0.72–0.85) | <0.001 | 0.78 (0.72–0.85) | <0.001 |
| BMI (kg/m^2^) | 1.15 (1.14–1.16) | <0.001 | 1.15 (1.14–1.16) | <0.001 |
| Smoking status (ref. Never smoker) |  | 0.022 |  | 0.019 |
| Ex-smoker | 1.02 (0.92–1.13) | 0.704 | 1.02 (0.92–1.13) | 0.680 |
| Current smoker | 1.15 (1.03–1.28) | 0.010 | 1.15 (1.04–1.28) | 0.009 |
| Physical activity (ref. No) | 0.89 (0.84–0.94) | <0.001 | 0.89 (0.84–0.94) | <0.001 |
| Sodium intake ≥2.0g/day (ref. <2.0g/day) | 1.10 (1.03–1.18) | 0.005 | 1.10 (1.03–1.19) | 0.007 |
| Potassium intake (g/day) | 0.96 (0.93–0.99) | 0.016 | 0.96 (0.93–0.99) | 0.015 |
| Interaction: sodium intake ≥2.0g/day, PRS**_DBP_** (ref. Sodium <2.0g/day, PRS**_DBP_**) |  | 0.199 |  |  |
| Sodium ≥2.0g/day, PRS**_DBP_** | 1.04 (0.98–1.10) | 0.199 |  |  |
| Interaction: sodium intake, PRS**_DBP_** (ref. Sodium <2.0g/day, Middle) |  |  |  | 0.316 |
| Sodium intake ≥2.0g/day, Bottom 10% |  |  | 0.88 (0.71–1.09) | 0.231 |
| Sodium intake ≥2.0g/day, Top 10% |  |  | 1.07 (0.90–1.28) | 0.425 |

BMI, body mass index; CI, confidence interval; DBP, diastolic blood pressure; OR, odds ratio; PRS, polygenic risk score; SBP, systolic blood pressure; SD, standard deviation

Multivariable logistic regression, adjusted for PRS**_SBP_**, PRS**_DBP,_** age, sex, BMI, smoking status, physical activity, sodium intake, potassium intake, interaction of sodium intake and PRS**_DBP_**, and the first 10 principal components.

**Supplementary Table 7.** **Odds ratio for incident hypertension according to PRS, sodium and potassium intake, and their interaction**

| **Variables** | **PRS (continuous variable)** | | **PRS (categorial variable)** | | | |
| --- | --- | --- | --- | --- | --- | --- |
|  | **OR (95% CI)** | ***P*** | | **OR (95% CI)** | ***P*** |  |
| PRS_SBP_ (units of SD) | 1.23 (1.18–1.28) | <0.001 | |  |  |  |
| PRS**_SBP_** (ref. Middle) |  |  | |  | <0.001 |  |
| Bottom 10% |  |  | | 0.70 (0.63–0.79) | <0.001 |  |
| Top 10% |  |  | | 1.37 (1.25–1.51) | <0.001 |  |
| PRS**_DBP_** (units of SD) | 1.05 (1.01–1.09) | 0.021 | |  |  |  |
| PRS**_DBP_** (ref. Middle) |  |  | |  | <0.001 |  |
| Bottom 10% |  |  | | 0.84 (0.75–0.94) | 0.003 |  |
| Top 10% |  |  | | 1.27 (1.15–1.39) | <0.001 |  |
| Age (year) | 1.04 (1.04–1.04) | <0.001 | | 1.04 (1.03–1.04) | <0.001 |  |
| Sex, female (ref. male) | 0.79 (0.72–0.86) | <0.001 | | 0.79 (0.72–0.86) | <0.001 |  |
| BMI (kg/m^2^) | 1.15 (1.14–1.16) | <0.001 | | 1.15 (1.14–1.16) | <0.001 |  |
| Smoking status (ref. Never smoker) |  | 0.029 | |  | 0.025 |  |
| Ex-smoker | 1.02 (0.92–1.13) | 0.680 | | 1.02 (0.92–1.13) | 0.658 |  |
| Current smoker | 1.14 (1.03–1.27) | 0.013 | | 1.15 (1.04–1.28) | 0.011 |  |
| Physical activity (ref. No) | 0.90 (0.85–0.95) | <0.001 | | 0.90 (0.85–0.95) | <0.001 |  |
| Sodium intake (g/day) | 1.07 (1.03–1.11) | <0.001 | | 1.06 (1.02–1.10) | <0.001 |  |
| Potassium intake (g/day) | 0.90 (0.85–0.96) | <0.001 | | 0.90 (0.85–0.96) | <0.001 |  |
| Interaction: sodium intake, potassium intake | 1.00 (1.00–1.01) | 0.419 | | 1.00 (1.00–1.01) | 0.385 |  |

BMI, body mass index; CI, confidence interval; DBP, diastolic blood pressure; OR, odds ratio; PRS, polygenic risk score; SBP, systolic blood pressure; SD, standard deviation

Multivariable logistic regression, adjusted for PRS**_SBP_**, PRS**_DBP,_** age, sex, BMI, smoking status, physical activity, sodium intake, potassium intake, interaction of sodium and potassium intake, and the first 10 principal components.

**Supplementary Table 8. Odds ratio of dietary potassium intake for incident hypertension according to subgroups**

| **Subgroup** | **N** | **Multivariable** | |
| --- | --- | --- | --- |
|  |  | **OR (95% CI)** | ***P*** |
| **PRS_SBP_** |  |  |  |
| Bottom 10% | 4,136 | 0.98 (0.88–1.08) | 0.649 |
| Middle | 33,080 | 1.00 (0.97–1.03) | 0.845 |
| Top 10% | 4,135 | 0.98 (0.91–1.06) | 0.633 |
| **PRS_DBP_** |  |  |  |
| Bottom 10% | 4,136 | 0.96 (0.87–1.06) | 0.408 |
| Middle | 33,080 | 1.00 (0.97–1.03) | 0.831 |
| Top 10% | 4,135 | 1.02 (0.95–1.09) | 0.638 |
| **Age** |  |  |  |
| <55 years old | 24,517 | 0.99 (0.96–1.03) | 0.717 |
| ≥55 years old | 16,834 | 0.99 (0.95–1.02) | 0.451 |
| **Sex** |  |  |  |
| Male | 13,097 | 1.00 (0.96–1.04) | 0.988 |
| Female | 28,254 | 1.00 (0.97–1.03) | 0.952 |
| **BMI** |  |  |  |
| <18.5 kg/m^2^ | 870 | 0.94 (0.68–1.29) | 0.698 |
| 18.5-24.9 kg/m^2^ | 29,320 | 0.97 (0.94–1.01) | 0.091 |
| ≥25.0 kg/m^2^ | 11,155 | 1.01 (0.97–1.05) | 0.652 |
| **Smoking status** |  |  |  |
| Never smoker | 30,964 | 1.00 (0.97–1.04) | 0.823 |
| Ex-smoker | 5,667 | 1.04 (0.97–1.11) | 0.288 |
| Current smoker | 4,583 | 0.93 (0.86–1.00) | 0.051 |
| **Alcohol drinking** |  |  |  |
| No | 23,427 | 1.01 (0.98–1.05) | 0.523 |
| Yes | 17,761 | 0.98 (0.94–1.02) | 0.361 |
| **Physical activity** |  |  |  |
| No | 19,601 | 0.99 (0.95–1.03) | 0.551 |
| Yes | 21,609 | 1.02 (0.98–1.06) | 0.293 |
| **DM** |  |  |  |
| No | 38,585 | 1.01 (0.98–1.03) | 0.702 |
| Yes | 2,766 | 0.93 (0.84–1.02) | 0.120 |

BMI, body mass index; CI, confidence interval; DBP, diastolic blood pressure; OR, odds ratio; PRS, polygenic risk score; SBP, systolic blood pressure; SD, standard deviation

Multivariable logistic regression, adjusted for age, sex, potassium intake (continuous variable, g/day).
